# The CaMKII/NMDAR complex as a molecular memory

**DOI:** 10.1186/1756-6606-6-10

**Published:** 2013-02-14

**Authors:** Magdalena Sanhueza, John Lisman

**Affiliations:** 1 Department of Biology, Faculty of Sciences, University of Chile, Las Palmeras 3425, Santiago, 7800024, Chile; 2 Volen Center for Complex Systems, Biology Department, Brandeis University, 415 South Street, MS 008, Waltham, MA 02454-9110, USA

## Abstract

CaMKII is a major synaptic protein that is activated during the induction of long-term potentiation (LTP) by the Ca^2+^ influx through NMDARs. This activation is required for LTP induction, but the role of the kinase in the maintenance of LTP is less clear. Elucidating the mechanisms of maintenance may provide insights into the molecular processes that underlie the stability of stored memories. In this brief review, we will outline the criteria for evaluating an LTP maintenance mechanism. The specific hypothesis evaluated is that LTP is maintained by the complex of activated CaMKII with the NMDAR. The evidence in support of this hypothesis is substantial, but further experiments are required, notably to determine the time course and persistence of complex after LTP induction. Additional work is also required to elucidate how the CaMKII/NMDAR complex produces the structural growth of the synapse that underlies late LTP. It has been proposed by Frey and Morris that late LTP involves the setting of a molecular tag during LTP induction, which subsequently allows the activated synapse to capture the proteins responsible for late LTP. However, the molecular processes by which this leads to the structural growth that underlies late LTP are completely unclear. Based on known binding reactions, we suggest the first molecularly specific version of tag/capture hypothesis: that the CaMKII/NMDAR complex, once formed, serves as a tag, which then leads to a binding cascade involving densin, delta-catenin, and N-cadherin (some of which are newly synthesized). Delta-catenin binds AMPA-binding protein (ABP), leading to the LTP-induced increase in AMPA channel content. The addition of postsynaptic N-cadherin, and the complementary increase on the presynaptic side, leads to a trans-synaptically coordinated increase in synapse size (and more release sites). It is suggested that synaptic strength is stored stably through the combined actions of the CaMKII/NMDAR complex and N-cadherin dimers. These N-cadherin pairs have redundant storage that could provide informational stability in a manner analogous to the base-pairing in DNA.

## 

CaMKII is a highly abundant brain protein concentrated in the postsynaptic density (PSD) and is strongly implicated in LTP (reviewed in [1]). During the induction of LTP, Ca^2+^ enters through the NMDAR and binds to calmodulin [2]. Calmodulin then activates CaMKII, which phosphorylates the GluA1 subunits of AMPARs and an auxiliary subunit of AMPARs, stargazin. The first reaction increases the conductance of AMPARs [3,4]; the second allows more AMPARs to be bound into the synapse by PSD-95 [5,6]. Together, these processes provide a mechanistic explanation for the early phase of LTP (approximately the first 30–60 minutes). Later phases of LTP appear to require different mechanisms, and it is these mechanisms that maintain LTP for the long periods required for memory storage. The hypothesis that we will evaluate in this review is that late LTP is maintained by the complex of CaMKII with the NMDAR. Evidence relevant to the following criteria will be summarized:

1. LTP induction should cause a persistent increase in the CaMKII/NMDAR complex.

2. Inhibiting formation of the CaMKII/NMDAR complex should block LTP induction.

3. Decreasing the amount of CaMKII/NMDAR complex after LTP induction should reverse LTP.

4. A component of *basal* transmission should be reversed by reducing the basal CaMKII/NMDAR complex.

5. There must be a mechanism by which CaMKII/NMDAR complex can produce potentiation, specifically the trans-synaptic structural processes that underlie late LTP.

## LTP induction should cause a persistent increase in the CaMKII/NMDAR complex

The initial evidence that CaMKII could interact with the NMDAR came from *in vitro* experiments showing that a fragment of GluN2B is a substrate for purified CaMKII (at 1303) [7]. It was noted that the binding affinity of GluN2B was much higher than for other CaMKII substrates. Subsequent experiments showed that CaMKII could form a tight complex with NMDAR and that the complex was present in living cells, as identified by crosslinking or coimmunoprecipitation (co-IP) [8-10]. Importantly, the amount of complex was increased substantially by stimulation that elevated intracellular Ca^2+^ and thereby activated CaMKII.

A critical assumption of the proposed model is that LTP produces an increase in the CaMKII/NMDAR complex that is persistent for the duration of LTP. This assumption remains to be tested directly, but there is relevant evidence. Stimulation of cultured neurons with glutamate/glycine (a form of chemical LTP) can trigger persistent translocation of CaMKII that requires kinase binding to GluN2B at what is termed the T-site [11]. Importantly, the persistently translocated CaMKII is phosphorylated at T286 [12]. Furthermore, chemical LTP produces translocation of CaMKII to the PSD, as visualized by electron microscopy; this persists for at least 1 hr after LTP induction, suggesting that there is persistent formation of the CaMKII/NMDAR complex [13,14].

The binding of CaMKII to the NMDAR requires an activated open form of the kinase. Therefore, the recent optical experiments [15] demonstrating that CaMKII is only transiently activated after LTP induction (~1 minute) could suggest that the CaMKII/NMDAR complex is also transient. However, there are two reasons to doubt the generality of this conclusion. First, recent measurements indicate that the fraction of CaMKII subunits directly bound to the NMDAR and thereby locked in the open confirmation [16] is a small fraction of the total CaMKII in spines and may be difficult to detect optically [17]. Second, the optical measurements of CaMKII activation were made under conditions that did not evoke late LTP [18], raising the possibility that future measurement under conditions that did induce late LTP would evoke a detectable persistent component, as seen with induction protocols based on tetanic stimulation [19]. In any case, given that the key question is the duration of the LTP-induced increase in CaMKII/NMDAR complex, what is needed is a method (possibly based on FRET) that would make it possible to directly monitor the kinetics of complex formation and persistence.

## Inhibiting formation of the CaMKII/NMDAR complex should block LTP induction

To examine the functional role of complex formation, Barria and Malinow [20] overexpressed a form of GluN2B having mutations near the CaMKII phosphorylation site (R1300Q and S1303D) that strongly interferes with binding [21]. LTP was examined using a low-frequency pairing protocol. There was a significant (but not complete) block of potentiation in the first 10 minutes after LTP induction; at later times, the block was complete.

Related experiments were conducted in hippocampal slices from knockin (KI) mice in which CaMKII-GluN2B binding was impaired by the mutations L1298A and R1300Q [22]. In these mice, the basal CaMKII/NMDAR complex was reduced by about 40%. Activity-dependent formation of complex in cell cultures was more strongly reduced. LTP induced by high-frequency stimulation in slice experiments was reduced, but only by about 50%. It is unclear whether the smaller reduction than in the Barria/Malinow experiments is due to a slightly different mutation, to the difference in animal age, or to a difference in the LTP induction protocol.

Another method for interfering with the CaMKII/NMDAR complex utilized transgenic mice in which the whole C-terminal GluN2B fragment could be induced [23]. The LTP induced by high-frequency stimulation was reduced by about 50%. It is unclear whether this reduction was due to inhibiting CaMKII activity or to inhibiting the formation of the CaMKII/NMDAR complex.

A complementary experiment would be to determine the effects of increasing the CaMKII/NMDAR complex. Overexpression of activated CaMKII holoenzyme increases synaptic strength and spine size, provided that phosphorylation of T305/306 is prevented [24]. It would be expected that this form of holoenzyme would increase the CaMKII/NMDAR complex and synapse size, but this has not been directly tested.

## Decreasing the amount of CaMKII/NMDAR complex after LTP induction should reverse LTP

An important tool has been the development of a related group of peptides (CN27; CN21; CN19) that block the formation of the CaMKII/NMDAR complex *in vitro*[25]. This peptide family derives from a fragment of an endogenous CaMKII inhibitor protein, CaMKIIN [26]. This protein strongly binds to a site on CaMKII (the T-site) in addition to the catalytic site to which other peptide inhibitors mainly bind (these are based on the sequence of the CaMKII regulatory domain). It is to the T-site that the NMDAR itself binds.

**Figure 1:**
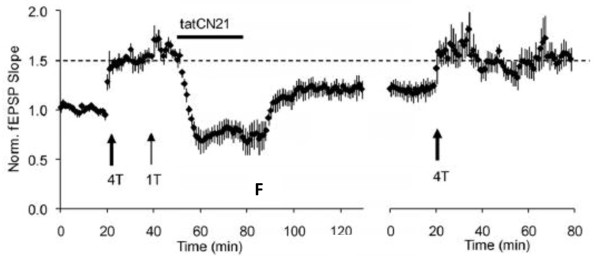
**TatCN21 reverses LTP. **fEPSPs recorded from the CA1 region of a hippocampal slice. At 20 min, LTP was induced by four tetani. This LTP was saturated, as evidenced by lack of further potentiation when an additional tetanus was given at 40 min. tatCN21 was then applied for 30 min. Upon removal, there was partial recovery, but also a non-recoverable component that demonstrates LTP reversal. To verify that saturated LTP had indeed been reversed, an additional tetanus was given (right), and this reinduced LTP. In control experiments without tatCN21 application, LTP could not be reinduced.

Application of CN21 can reverse LTP in hippocampal slices [27], as shown in Figure 1. An action of this kind would be consistent with an effect on either LTP expression or maintenance processes. An experiment that distinguishes between these possibilities is to remove the peptide; if the effect was on expression, LTP should recover. Figure 1 shows that LTP does not return. It could be argued that this lack of return resulted from damage; however, the ability to then reinduce LTP argues against this possibility. Thus, CN21 appears to reset a molecular switch that controls LTP maintenance.

Several controls point to the fact that CN21 works in slices to reduce the CaMKII/NMDAR complex, as expected from *in vitro* work. First, the peptide reduced the amount of basal complex, as measured by co-IP (basal transmission was also reduced; see next section). If somewhat lower concentrations of CN21 were used (5 μM instead of 20 μM), there was no reduction in the CaMKII/NMDAR complex and no persistent reduction in basal transmission. Second, the peptide produced a small but statistically significant reduction in the amount of CaMKII bound in spines. Third, CN21 produced a large reduction in the activity-dependent increase of CaMKII in the PSD [28].

The ability of CN compounds to rapidly reverse LTP is not shared by other peptides that primarily inhibit the CaMKII catalytic site [29,30]. This suggests that the reversal is due to interference with structural rather than enzymatic reactions. This could mean that there is no role for enzymatic activity (including autophosphorylation-induced autonomous activity [16]) during LTP maintenance. Alternatively, the effects of inhibiting such activity may require many hours to develop and thus have not been seen in the much shorter experiments performed thus far. Indeed, experiments have shown that although phosphatase is present in the PSD, there is negligible dephosphorylation of CaMKII T286 within 1 hr [31].

## A component of *basal* transmission should be reversed by reducing the basal CaMKII/NMDAR complex

LTP has been observed *in vivo* during learning [32,33]. Moreover, synapses that have undergone learning-dependent LTP cannot then undergo LTP by electrical stimulation. This occlusion suggests that learning has saturated the normal LTP process. It would thus be expected that at least part of basal transmission, as measured in hippocampal slices, is the result of LTP-like events that occurred while the animal was alive. This inference is confirmed by analysis of unitary responses in the slice: the stronger the basal transmission, the smaller the magnitude of the LTP that can be induced [34,35].

Based on the data outline in the above paragraph, a strong expectation is that an agent that affects a synaptic memory mechanism should reduce basal transmission. Consistent with this, CN compounds depress basal transmission [27]. Moreover, this depression persists after removal of the CN compound, and the LTP that can then be induced is larger than if a scrambled control peptide or no peptide had been applied.

CN-induced depression is observed for drug concentrations necessary to disrupt basal CaMKII-NMDAR interaction in the slices, but not for lower concentrations causing only kinase inhibition [27]. Moreover, this type of depression is different from known forms of activity-dependent long-term depression (LTD), and it does not require Ca^2+^ influx, protein synthesis, or degradation [36]. The CN-induced persistent depression was barely detected in very young animals (P7-P10; [36]) for which CaMKII content at synapses is comparatively much lower [37,38]. Taken together, these findings strongly suggest that CN-induced depression of basal transmission is caused by breakdown of the CaMKII-NMDAR interaction at the synapse. Perhaps inconsistent with this conclusion is the fact that basal transmission was not reduced when the basal level of CaMKII/NMDAR complex was reduced by knockin of NMDAR mutations [22]. However, it is difficult to interpret such knockin experiments because known homeostatic processes could have normalized transmission over the lifetime of the animal.

## There must be a mechanism by which CaMKII/NMDAR complex can lead to the trans-synaptic structural growth that underlies late LTP

LTP has an early phase that does not involve synapse growth and does not require protein synthesis; this is followed within ~1 hr by late LTP, which involves synapse growth and requires protein synthesis [39,40]. An important concept is the tag and capture model [41]. According to this model, strong stimulation leads to “tagging” of the stimulated synapse by addition of a protein; this then serves to capture newly synthesized proteins, leading to late LTP at the activated synapse. Evidence suggests that CaMKII is the tag [42,43].

Relatively little is known about how proteins produce the synaptic growth that underlies late LTP. One area of progress has established a key role for N-cadherin, a homophilic adhesion molecule that forms a trans-synaptic linkage between the presynaptic and postsynaptic sides of the synapse. Pharmacological experiments, gene knockout experiments, conditional knockout experiments, and RNAi experiments all show that N-cadherin is required for late LTP (but not for early LTP) [44-46]. Furthermore, LTP induction causes synthesis of N-cadherin and its insertion into the synapse [44]. Finally, electron microscopy shows that overexpression of N-cadherin can increase synapse size [47]. Thus, although there are many forms of adhesion molecules at synapses, N-cadherin appears to be of central importance in late LTP.

**Figure 2:**
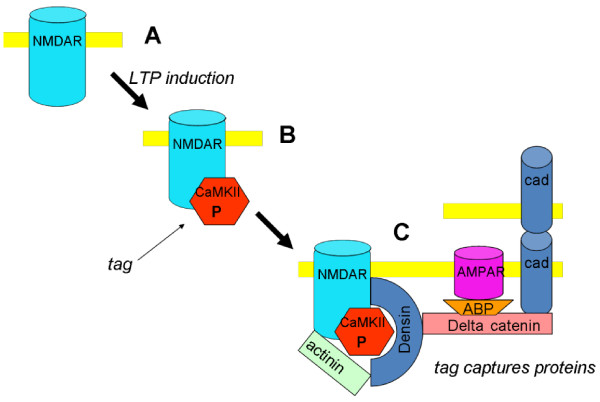
**Working hypothesis for how formation of CaMKII/NMDAR complex during LTP induction leads to subsequent binding reactions necessary for late LTP. A**, Before LTP induction (some NMDA channels have no CaMKII bound). **B**, During LTP induction, CaMKII is activated and forms a persistent complex with the NMDA channel, thus forming a tag. **C**, This serves as a structural seed for the gradual capture of densin, delta-catenin, ABP, and N-cadherin (cad). The addition of ABP provides additional anchoring sites for AMPA channels, thereby strengthening transmission. The binding of additional N-cadherin (which is synthesized in response to activity) enlarges the synapse both presynaptically and postsynaptically.

**Figure 3:**
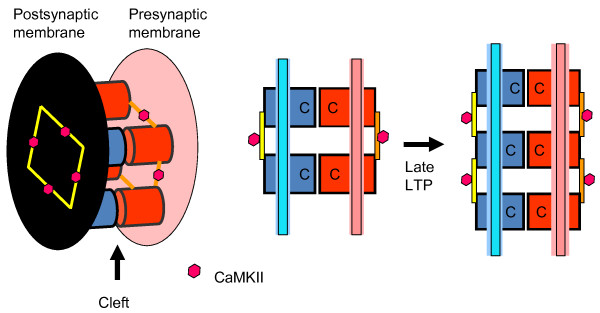
**Model of trans-synaptic growth.** Presynaptic (red) and postsynaptic (blue) cadherins (C) form homophilic bonds in the synaptic cleft (analogous to base-pairing in DNA). Cadherins are crosslinked by proteins in the postsynaptic density and presynaptic grid (yellow, orange), including CaMKII (red hexagons), which are analogous to the backbone of the DNA strands. Middle 2-D picture emphasizes analogy to DNA. Synapse size and strength are determined by the number of N-cadherin dimers. This number is redundantly stored by presynaptic and postsynaptic cadherin arrays and increases during late LTP.

There have been no previous suggestions about the cascade of biochemical events by which LTP induction results in the incorporation of N-cadherin into the synapse. Figure 2 shows a working hypothesis based on known binding interactions. It is proposed that the CaMKII/NMDAR complex acts as a structural seed for a series of binding reactions that gradually increase synapse size and strength, thereby accounting for late LTP. The following known binding interactions could be involved: densin-180 (and actinin) binds to the CaMKII in the CaMKII/NMDAR complex [48,49]; delta-catenin binds to densin [50]; and N-cadherin binds to delta-catenin [51]. As noted above, overexpression of N-cadherin leads to synapse growth. We thus posit that the addition of N-cadherin to the synapse during late LTP similarly leads to synapse growth. An important constraint on any model of synapse growth is that it must be trans-synaptically coordinated, leading to precise registration of the edges of the presynaptic grid and postsynaptic density [52]. The role of the N-cadherin trans-synaptic dimers in organizing synaptic growth provides a simple explanation of this coordination (Figure 3).

Physiological analysis suggests that late LTP involves both addition of AMPA receptors and an increase in presynaptic release sites [53,54]. It is thus of interest that delta-catenin binds to AMPA-binding protein (ABP) [55] and may thereby increase the AMPA channel (notably GluA2) content of the synapse [56-58]. N-cadherin can also bind directly to GluA2 [59], see also [60]. There is relatively little that can be said about the presynaptic changes that lead to enhanced vesicle release. It is known that overexpression of postsynaptic N-cadherin affects presynaptic function [61], but how this happens is unclear. Perhaps presynaptic N-cadherin is brought into the synapse simply by binding to postsynaptic N-cadherin. Alternatively, insertion of presynaptic N-cadherin might depend on the presynaptic activation of CaMKII [62], translocation of CaMKII to the presynaptic grid [63], and a protein linkage to N-cadherin [51] analogous to the postsynaptic linkage described in Figure 2.

It is noteworthy that Figure 3 suggests a limited analogy to DNA: just as in DNA, there are two crosslinked backbone structures attached to elements that link the two backbone structures by a pairing rule (in DNA, the rule is heterologous; here, it is homologous). The existence of two backbone structures provides redundant information storage. Thus the N-cadherin dimers could contribute to informational stability, just as base-pairing does in DNA. Specifically, if one member of an N-cadherin dimer was lost in the process of turnover, the existence of the other member could serve as a basis for repair. In this way, the number of N-cadherin dimers could be stable despite turnover of N-cadherin. This stability, in turn, could help to stabilize the CaMKII/NMDAR complex, as described in the next paragraph.

Although we have posited that the CaMKII/NMDAR complex brings about the capture of the protein complex that includes actinin, densin, delta-catenin, and N-cadherin, the complex, once established, may be important for the stabilization of CaMKII/NMDAR complex during protein turnover. During turnover of CaMKII, phosphorylated CaMKII holoenzymes may be removed from the NMDAR, leaving a pocket formed by densin and actinin. This pocket is relatively stable because it is held there by other parts of the complex that include the N-cadherin dimmers (Figure 2). The phospho-CaMKII that left the pocket can be replaced by CaMKII that is either not phosphorylated (but with Ca^2+^/calmodulin bound) or weakly phosphorylated. There, CaMKII can bind to GluN2B [11] and will become autophosphorylated as a result of stimulation by actinin [64] and GluN2B [16] and because PSD CaMKII is protected from phosphatase [31] (probably because of the protein structure around bound CaMKII). This highly phosphorylated state then leads to tighter binding to the NMDAR [11]. In this way, the original tight complex of CaMKII bound to the NMDAR can be restored despite the molecular turnover.

This model provides an explanation for why overexpression of catalytically dead CaMKII can reverse memory (see next section). Specifically, this form (K42M) can bind to the NMDAR (albeit weakly), but not undergo the autophosphorylation necessary for tight binding to the NMDAR [11] and densin [65]. Weak binding to the NMDAR and densin may ultimately lead to dissociation of the entire complex, resulting in loss of LTP and memory.

Our model leads to testable predictions: that densin-180, delta-catenin, and N-cadherin are required for synapse growth and late LTP. As noted earlier, N-cadherin is required for late LTP (but not early LTP) and for synapse growth. Knockout of delta-catenin or densin does not block early LTP [58,66]; the effect on late LTP or synapse growth has not been tested.

## Effect of altering the CaMKII/NMDAR complex on behavioral tests of memory

Evidence that CaMKII signaling contributes strongly to the maintenance of nonspatial forms of memory has recently been published. One line of work comes from research on addiction in rats [67]. It was found that a persistent reversal of drug-induced sensitization could be produced by transient expression of a kinase-dead CaMKII mutant (K42M). In these experiments, animals were initially subjected to repetitive amphetamine exposure. Subsequently, transient virally mediated expression of K42M produced a persistent block of the normal long-lasting behavioral response to the drug (enhanced locomotion and drug self-administration). Because expression of K42M had ceased at the time of testing, the action of K42M must have been to reset a memory switch. In addition to the lack of catalytic activity and autophosphorylation, K42M displays an altered pattern of synaptic translocation in cultured neurons. While transient translocation is not affected [68,69], the strength of binding to the NMDAR is strongly decreased [21,70]. Thus, there is a reasonable basis for suspecting that K42M can erase a memory by acting as a dominant negative for stable CaMKII/NMDAR complex. A further indication that CaMKII is involved in the maintenance of behavioral memory comes from the experiments of [71]. They found that conditioned fear could be erased by transient post-learning inhibition of overexpressed CaMKII. However, the inhibition was animal-wide, so the location and physiological correlate of this effect are unclear.

Recent experiments have sought to test directly the role of the CaMKII/NMDAR complex in memory. To address this issue, [22] utilized a knockin mouse with mutations L1298A and R1300Q in GluN2B that interfere with that interferes with the formation of the CaMKII/NMDAR complex. A key finding was that spatial memory in the Morris water maze task, when measured at 1 or 3 days after the last training session, was greatly reduced (memory 1-2 hr after each training session was not reduced). Interpretation of the results is complicated by the fact that the mutation only produced a partial reduction in the basal CaMKII/NMDAR complex and only a ~50% reduction in LTP. It is thus possible that the learning that occurred in this task was due to this remaining ability of complex to form. It is also of interest to consider the possibility that CaMKII can form other complexes (i.e., not with NMDARs) that contribute to synaptic enhancement. Indeed, if presynaptic CaMKII is necessary for binding of N-cadherin and growth of the presynaptic active zone, the binding of CaMKII to a presynaptic protein, possibly Ca^2+^ channels [72], could contribute to presynaptic structural changes during LTP. A further question is whether memory maintenance can be reversed by interfering with the CaMKII/NMDAR complex after learning. Only one study thus far deals with this issue [73]. It was found that a CN peptide injected into the cingulate cortex was able to reverse a form of central pain. Importantly, induction of central pain produced a large increase in the amount of CaMKII/NMDAR complex in PSDs of the cingulate, and this increase was reversed by CN.

## The PKM-zeta alternative

It is noteworthy that a major alternative hypothesis [74], that memory is stored by a persistent increase in PKM-zeta, has substantial weaknesses, many of which have only recently become apparent. A critical test of the effect of PKM-zeta to reverse LTP maintenance has not been done. While it was shown that LTP in the slice could be reduced by ZIP, it was not shown that this reduction persisted after removal of ZIP [75]. In different experiments in which ZIP was transiently injected *in vivo*, the drug was still present 2 hr after injection, as shown by immunochemistry; no removal of drug was demonstrated [76]. Thus, the existing experiments do not distinguish between effects on LTP maintenance and effects on LTP expression. Furthermore, recent work casts strong doubt on whether ZIP has its effect by an action on PKM-zeta [1,77,78]. Importantly, recent work from the Sacktor laboratory demonstrates that the control peptide (scrambled ZIP) can also inhibit PKM-zeta and is only three times less effective than ZIP [79]. With such small differences in efficacy, it would take very precise administration of these peptides to produce a differential effect, but the concentrations used in *in vivo* experiments were highly imprecise (several orders of magnitude above the K_d_) [1]. Thus, the differential effects on ZIP and scrambled ZIP are probably due to an effect on some target other than PKM-zeta. Finally, knockout of this enzyme did not affect late LTP, memory, or the effect of ZIP [80] (but see [81]). Thus, the evidence that PKM-zeta is the molecular basis of memory is not compelling.

## Conclusions

The CaMKII/NMDAR complex is a promising candidate as the molecular basis of memory storage. Although there is increasing evidence that CaMKII and its binding partners form a molecular memory, additional work is required to prove this hypothesis. In particular, methods for studying the formation and persistence of the complex during actual LTP are needed. Furthermore, much additional work is required at the behavioral level to test the role of the CaMKII and the CaMKII/NMDAR complex in the persistence of memory. Most of the work on this complex has been done in the hippocampus Thus, it will be of particular importance to determine whether interference with the complex in the hippocampus can reverse a hippocampal-dependent spatial memory.

An important unresolved question is whether the importance of CaMKII/NMDAR complex in LTP maintenance, as demonstrated in Figure 1, will be specific for CA1 or more generally applicable to synapses. One perspective on this question comes from analysis of the PSDs, which are generally isolated from whole brains. Structural analysis shows that these PSDs are nearly tight-packed with CaMKII [82]. Given that these PSDs are representative of the whole brain, it seems likely that a critical role for CaMKII will be a widespread property. It has become increasingly clear that an important aspect of activity-dependent synaptic plasticity is the making and breaking of synaptic connections. Recent work shows that the ability of activity to stabilize synaptic connections is dependent on the CaMKII/NMDAR complex [83]. Thus, similar mechanisms may be involved in changes in synaptic strength and in stabilization of synaptic connections.

## Competing interests

The authors declare that they have no competing interests.

## Authors' contributions

JL and MS contributed equally to writing this review. Both authors read and approved the final manuscript.

## References

[B1] Lisman J Yasuda R Raghavachari S Mechanisms of CaMKII action in long-term potentiation Nat Rev Neurosci 2012 13 3 169 182 22334212 10.1038/nrn3192 PMC4050655

[B2] Faas G Calmodulin as a direct detector of Ca2+ signals Nat Neurosci 2011 14 3 301 304 10.1038/nn.2746 21258328 PMC3057387

[B3] Benke TA Modulation of AMPA receptor unitary conductance by synaptic activity Nature 1998 393 6687 793 797 10.1038/31709 9655394

[B4] Kristensen AS Mechanism of Ca2+/calmodulin-dependent kinase II regulation of AMPA receptor gating Nat Neurosci 2011 14 6 727 35 10.1038/nn.2804 21516102 PMC3102786

[B5] Tomita S Bidirectional synaptic plasticity regulated by phosphorylation of stargazin-like TARPs Neuron 2005 45 2 269 77 10.1016/j.neuron.2005.01.009 15664178

[B6] Opazo P CaMKII triggers the diffusional trapping of surface AMPARs through phosphorylation of stargazin Neuron 2010 67 2 239 252 10.1016/j.neuron.2010.06.007 20670832

[B7] Omkumar RV Identification of a phosphorylation site for calcium/calmodulindependent protein kinase II in the NR2B subunit of the N-methyl-D-aspartate receptor J Biol Chem 1996 271 49 31670 31678 10.1074/jbc.271.49.31670 8940188

[B8] Strack S Colbran RJ Autophosphorylation-dependent targeting of calcium/ calmodulin-dependent protein kinase II by the NR2B subunit of the N-methyl- D-aspartate receptor J Biol Chem 1998 273 33 20689 20692 10.1074/jbc.273.33.20689 9694809

[B9] Gardoni F Calcium/calmodulin-dependent protein kinase II is associated with NR2A/B subunits of NMDA receptor in postsynaptic densities J Neurochem 1998 71 4 1733 1741 9751209 10.1046/j.1471-4159.1998.71041733.x

[B10] Leonard AS Calcium/calmodulin-dependent protein kinase II is associated with the N-methyl-D-aspartate receptor Proc Natl Acad Sci USA 1999 96 6 3239 3244 10.1073/pnas.96.6.3239 10077668 PMC15926

[B11] Bayer KU Transition from reversible to persistent binding of CaMKII to postsynaptic sites and NR2B J Neurosci 2006 26 4 1164 1174 10.1523/JNEUROSCI.3116-05.2006 16436603 PMC2890238

[B12] Appleby VJ LTP in hippocampal neurons is associated with a CaMKII-mediated increase in GluA1 surface expression J Neurochem 2011 116 4 530 543 10.1111/j.1471-4159.2010.07133.x 21143596

[B13] Otmakhov N Persistent accumulation of calcium/calmodulin-dependent protein kinase II in dendritic spines after induction of NMDA receptor-dependent chemical long-term potentiation The Journal of neuroscience: the official journal of the Society for Neuroscience 2004 24 42 9324 31 10.1523/JNEUROSCI.2350-04.2004 15496668 PMC6730088

[B14] Zhang YP Holbro N Oertner TG Optical induction of plasticity at single synapses reveals input-specific accumulation of alphaCaMKII Proc Natl Acad Sci USA 2008 105 33 12039 44 10.1073/pnas.0802940105 18697934 PMC2575337

[B15] Lee SJ Yasuda R Spatiotemporal Regulation of Signaling in and out of Dendritic Spines: CaMKII and Ras The open neuroscience journal 2009 3 117 127 10.2174/1874082000903020117 20463853 PMC2867484

[B16] Bayer KU Interaction with the NMDA receptor locks CaMKII in an active conformation Nature 2001 411 6839 801 5 10.1038/35081080 11459059

[B17] Feng B Raghavachari S Lisman J Quantitative estimates of the cytoplasmic, PSD, and NMDAR-bound pools of CaMKII in dendritic spines Brain Res 2011 1419 46 52 21925648 10.1016/j.brainres.2011.08.051 PMC3196057

[B18] Tanaka J-I Protein synthesis and neurotrophin-dependent structural plasticity of single dendritic spines Science 2008 319 5870 1683 1687 10.1126/science.1152864 18309046 PMC4218863

[B19] Fukunaga K Long-term potentiation is associated with an increased activity of Ca2+/calmodulin-dependent protein kinase II J Biol Chem 1993 268 11 7863 7 8385124

[B20] Barria A Malinow R NMDA receptor subunit composition controls synaptic plasticity by regulating binding to CaMKII Neuron 2005 48 2 289 301 10.1016/j.neuron.2005.08.034 16242409

[B21] Strack S McNeill RB Colbran RJ Mechanism and regulation of calcium/calmodulin-dependent protein kinase II targeting to the NR2B subunit of the N-methyl-D-aspartate receptor J Biol Chem 2000 275 31 23798 806 10.1074/jbc.M001471200 10764765

[B22] Halt A CaMKII binding to GluN2B is critical during memory consolidation EMBO J 2012 31 5 1203 1216 10.1038/emboj.2011.482 22234183 PMC3297991

[B23] Zhou Y Interactions between the NR2B receptor and CaMKII modulate synaptic plasticity and spatial learning J Neurosci 2007 27 50 13843 13853 10.1523/JNEUROSCI.4486-07.2007 18077696 PMC6673634

[B24] Pi HJ CaMKII control of spine size and synaptic strength: role of phosphorylation states and nonenzymatic action Proc Natl Acad Sci USA 2010 107 32 14437 42 10.1073/pnas.1009268107 20660727 PMC2922610

[B25] Vest RS Dual mechanism of a natural CaMKII inhibitor Mol Biol Cell 2007 18 12 5024 33 10.1091/mbc.E07-02-0185 17942605 PMC2096578

[B26] Chang BH Mukherji S Soderling TR Characterization of a calmodulin kinase II inhibitor protein in brain Proc Natl Acad Sci USA 1998 95 18 10890 5 10.1073/pnas.95.18.10890 9724800 PMC27991

[B27] Sanhueza M Role of the CaMKII/NMDA receptor complex in the maintenance of synaptic strength J Neurosci 2011 31 25 9170 9178 10.1523/JNEUROSCI.1250-11.2011 21697368 PMC3138556

[B28] Dosemeci A The effect of CaMKII inhibitor tatCN21 on the re-distribution of CaMKII and SynGAP in hippocampal neurons under excitatory conditions. SFN Program#/Poster#: 43.15/C53. In Society for Neuroscience 2012 New Orleans

[B29] Otmakhov N Griffith LC Lisman JE Postsynaptic inhibitors of calcium/calmodulin-dependent protein kinase type II block induction but not maintenance of pairing-induced long-term potentiation J Neurosci 1997 17 14 5357 5365 9204920 10.1523/JNEUROSCI.17-14-05357.1997 PMC6793827

[B30] Chen HX Is persistent activity of calcium/calmodulin-dependent kinase required for the maintenance of LTP? J Neurophysiol 2001 85 4 1368 1376 11287461 10.1152/jn.2001.85.4.1368

[B31] Mullasseril P A structural mechanism for maintaining the ‘on-state’ of the CaMKII memory switch in the post-synaptic density J Neurochem 2007 103 1 357 64 17877639 10.1111/j.1471-4159.2007.04744.x PMC2665908

[B32] Gruart A Munoz MD Delgado-Garcia JM Involvement of the CA3-CA1 synapse in the acquisition of associative learning in behaving mice The Journal of neuroscience: the official journal of the Society for Neuroscience 2006 26 4 1077 87 10.1523/JNEUROSCI.2834-05.2006 16436593 PMC6674570

[B33] Whitlock JR Learning induces long-term potentiation in the hippocampus Science 2006 313 5790 1093 7 10.1126/science.1128134 16931756

[B34] Liao D Jones A Malinow R Direct measurement of quantal changes underlying long-term potentiation in CA1 hippocampus Neuron 1992 9 6 1089 1097 10.1016/0896-6273(92)90068-O 1334418

[B35] Debanne D Gahwiler BH Thompson SM Heterogeneity of synaptic plasticity at unitary CA3-CA1 and CA3-CA3 connections in rat hippocampal slice cultures The Journal of neuroscience: the official journal of the Society for Neuroscience 1999 19 24 10664 71 10594050 10.1523/JNEUROSCI.19-24-10664.1999 PMC6784957

[B36] Gouet C On the Mechanism of Synaptic Depression Induced by CaMKIIN, an Endogenous Inhibitor of CaMKII PLoS One 2012 7 11 e49293 10.1371/journal.pone.0049293 23145145 PMC3493544

[B37] Petralia RS Ontogeny of postsynaptic density proteins at glutamatergic synapses Mol Cell Neurosci 2005 29 3 436 52 10.1016/j.mcn.2005.03.013 15894489 PMC1414063

[B38] Swulius MT Structure and composition of the postsynaptic density during development J Comp Neurol 2010 518 20 4243 60 10.1002/cne.22451 20878786 PMC2948241

[B39] Bailey CH Kandel ER Synaptic remodeling, synaptic growth and the storage of long-term memory in Aplysia Prog Brain Res 2008 169 179 198 18394474 10.1016/S0079-6123(07)00010-6

[B40] Bourne JN Harris KM Coordination of size and number of excitatory and inhibitory synapses results in a balanced structural plasticity along mature hippocampal CA1 dendrites during LTP Hippocampus 2011 21 4 354 73 10.1002/hipo.20768 20101601 PMC2891364

[B41] Frey U Morris RG Synaptic tagging and long-term potentiation Nature 1997 385 6616 533 536 10.1038/385533a0 9020359

[B42] Garic A CaMKII activation is a novel effector of alcohol’s neurotoxicity in neural crest stem/progenitor cells J Neurochem 2011 118 4 646 57 10.1111/j.1471-4159.2011.07273.x 21496022 PMC3137720

[B43] Redondo RL Synaptic tagging and capture: differential role of distinct calcium/calmodulin kinases in protein synthesis-dependent long-term potentiation The Journal of neuroscience: the official journal of the Society for Neuroscience 2010 30 14 4981 9 10.1523/JNEUROSCI.3140-09.2010 20371818 PMC6632790

[B44] Bozdagi O Increasing numbers of synaptic puncta during late-phase LTP: N-cadherin is synthesized, recruited to synaptic sites, and required for potentiation Neuron 2000 28 1 245 59 10.1016/S0896-6273(00)00100-8 11086998

[B45] Bozdagi O Persistence of coordinated long-term potentiation and dendritic spine enlargement at mature hippocampal CA1 synapses requires N-cadherin The Journal of neuroscience: the official journal of the Society for Neuroscience 2010 30 30 9984 9 10.1523/JNEUROSCI.1223-10.2010 20668183 PMC2921177

[B46] Tang L Hung CP Schuman EM A role for the cadherin family of cell adhesion molecules in hippocampal long-term potentiation Neuron 1998 20 6 1165 75 10.1016/S0896-6273(00)80497-3 9655504

[B47] Mendez P N-cadherin mediates plasticity-induced long-term spine stabilization J Cell Biol 2010 189 3 589 600 10.1083/jcb.201003007 20440002 PMC2867305

[B48] Walikonis RS Densin-180 forms a ternary complex with the (alpha)-subunit of Ca2+/calmodulin-dependent protein kinase II and (alpha)-actinin The Journal of neuroscience: the official journal of the Society for Neuroscience 2001 21 2 423 33 11160423 10.1523/JNEUROSCI.21-02-00423.2001 PMC6763799

[B49] Robison AJ Multivalent Interactions of Calcium/Calmodulin-dependent Protein Kinase II with the Postsynaptic Density Proteins NR2B, Densin-180, and α-Actinin-2 J Biol Chem 2005 280 42 35329 35336 10.1074/jbc.M502191200 16120608

[B50] Izawa I Densin-180 interacts with delta-catenin/neural plakophilin-related armadillo repeat protein at synapses J Biol Chem 2002 277 7 5345 50 10.1074/jbc.M110052200 11729199

[B51] Brigidi GS Bamji SX Cadherin-catenin adhesion complexes at the synapse Curr Opin Neurobiol 2011 21 2 208 14 10.1016/j.conb.2010.12.004 21255999

[B52] Lisman JE Harris KM Quantal analysis and synaptic anatomy–integrating two views of hippocampal plasticity Trends Neurosci 1993 16 4 141 7 10.1016/0166-2236(93)90122-3 7682347

[B53] Lisman J The pre/post LTP debate Neuron 2009 63 3 281 284 10.1016/j.neuron.2009.07.020 19679068

[B54] Lisman J Raghavachari S A unified model of the presynaptic and postsynaptic changes during LTP at CA1 synapses Science’s signal transduction knowledge environment 2006 2006 356 re11 re11 10.1126/stke.3562006re11 17033044

[B55] Silverman JB Synaptic anchorage of AMPA receptors by cadherins through neural plakophilin-related arm protein-AMPA receptor-binding protein complexes J Neurosci 2007 27 32 8505 8516 10.1523/JNEUROSCI.1395-07.2007 17687028 PMC6672939

[B56] DeSouza S Differential Palmitoylation Directs the AMPA Receptor-Binding Protein ABP to Spines or to Intracellular Clusters J Neurosci 2002 22 9 3493 3503 11978826 10.1523/JNEUROSCI.22-09-03493.2002 PMC6758378

[B57] Ochiishi T Regulation of AMPA receptor trafficking by δ-catenin Mol Cell Neurosci 2008 39 4 499 507 10.1016/j.mcn.2008.06.002 18602475

[B58] Israely I Deletion of the neuron-specific protein delta-catenin leads to severe cognitive and synaptic dysfunction Current biology: CB 2004 14 18 1657 63 10.1016/j.cub.2004.08.065 15380068

[B59] Saglietti L Extracellular interactions between GluR2 and N-cadherin in spine regulation Neuron 2007 54 3 461 77 10.1016/j.neuron.2007.04.012 17481398

[B60] Zhou Z GluA2 (GluR2) regulates metabotropic glutamate receptor-dependent long-term depression through N-cadherin-dependent and cofilin-mediated actin reorganization The Journal of neuroscience: the official journal of the Society for Neuroscience 2011 31 3 819 33 10.1523/JNEUROSCI.3869-10.2011 21248105 PMC6632944

[B61] Vitureira N Differential control of presynaptic efficacy by postsynaptic N-cadherin and [beta]-catenin Nat Neurosci 2012 15 1 81 89 22138644 10.1038/nn.2995 PMC3245860

[B62] Lu FM Hawkins RD Presynaptic and postsynaptic Ca(2+) and CamKII contribute to long-term potentiation at synapses between individual CA3 neurons Proc Natl Acad Sci USA 2006 103 11 4264 9 10.1073/pnas.0508162103 16537519 PMC1449681

[B63] Shakiryanova D Differential control of presynaptic CaMKII activation and translocation to active zones The Journal of neuroscience: the official journal of the Society for Neuroscience 2011 31 25 9093 100 10.1523/JNEUROSCI.0550-11.2011 21697360 PMC3123710

[B64] Jalan-Sakrikar N Substrate-selective and calcium-independent activation of CaMKII by alpha-actinin J Biol Chem 2012 287 19 15275 83 10.1074/jbc.M112.351817 22427672 PMC3346149

[B65] Strack S Association of calcium/calmodulin-dependent kinase II with developmentally regulated splice variants of the postsynaptic density protein densin-180 J Biol Chem 2000 275 33 25061 4 10.1074/jbc.C000319200 10827168

[B66] Carlisle HJ Deletion of densin-180 results in abnormal behaviors associated with mental illness and reduces mGluR5 and DISC1 in the postsynaptic density fraction The Journal of neuroscience: the official journal of the Society for Neuroscience 2011 31 45 16194 207 10.1523/JNEUROSCI.5877-10.2011 22072671 PMC3235477

[B67] Loweth JA Persistent Reversal of Enhanced Amphetamine Intake by Transient CaMKII Inhibition The Journal of neuroscience: the official journal of the Society for Neuroscience 2013 33 4 1411 6 10.1523/JNEUROSCI.4386-13.2013 23345217 PMC3710147

[B68] Shen K Meyer T Dynamic control of CaMKII translocation and localization in hippocampal neurons by NMDA receptor stimulation Science 1999 284 5411 162 6 10.1126/science.284.5411.162 10102820

[B69] Yamagata Y Kinase-dead knock-in mouse reveals an essential role of kinase activity of Ca2+/calmodulin-dependent protein kinase IIalpha in dendritic spine enlargement, long-term potentiation, and learning J Neurosci 2009 29 23 7607 7618 10.1523/JNEUROSCI.0707-09.2009 19515929 PMC6665418

[B70] O’Leary H Nucleotides and phosphorylation bi-directionally modulate Ca2+/calmodulin-dependent protein kinase II (CaMKII) binding to the N-methyl-D-aspartate (NMDA) receptor subunit GluN2B J Biol Chem 2011 286 36 31272 81 10.1074/jbc.M111.233668 21768120 PMC3173099

[B71] Wang H Inducible protein knockout reveals temporal requirement of CaMKII reactivation for memory consolidation in the brain Proc Natl Acad Sci USA 2003 100 7 4287 92 10.1073/pnas.0636870100 12646704 PMC153085

[B72] Jiang X Modulation of CaV2.1 channels by Ca2+/calmodulin-dependent protein kinase II bound to the C-terminal domain Proc Natl Acad Sci USA 2008 105 1 341 346 10.1073/pnas.0710213105 18162541 PMC2224214

[B73] Li Y Phosphorylated CaMKII post-synaptic binding to NR2B subunits in the anterior cingulate cortex mediates visceral pain in visceral hypersensitive rats J Neurochem 2012 121 4 662 671 10.1111/j.1471-4159.2012.07717.x 22380661

[B74] Sacktor T Memory maintenance by PKMzeta – an evolutionary perspective Mol Brain 2012 5 1 31 31 10.1186/1756-6606-5-31 22986281 PMC3517905

[B75] Serrano P Yao Y Sacktor T Persistent phosphorylation by protein kinase Mzeta maintains late-phase long-term potentiation J Neurosci 2005 25 8 1979 1984 10.1523/JNEUROSCI.5132-04.2005 15728837 PMC6726070

[B76] Pastalkova E Storage of spatial information by the maintenance mechanism of LTP Science 2006 313 5790 1141 4 10.1126/science.1128657 16931766

[B77] Wu-Zhang A Cellular pharmacology of protein kinase Mζ (PKMζ) contrasts with its in vitro profile: implications for PKMζ as a mediator of memory J Biol Chem 2012 287 16 12879 12885 10.1074/jbc.M112.357244 22378786 PMC3339930

[B78] Lisman J Memory erasure by very high concentrations of ZIP may not be due to PKM-zeta Hippocampus 2012 22 3 648 649 10.1002/hipo.20980 21956821

[B79] Yao Y Matching biochemical and functional efficacies confirm ZIP as a potent competitive inhibitor of PKMζ in neurons Neuropharmacology 2013 64 37 44 22846225 10.1016/j.neuropharm.2012.07.018 PMC3445653

[B80] Volk LJ PKM-zeta is not required for hippocampal synaptic plasticity, learning and memory Nature 2013 493 7432 420 3 10.1038/nature11802 23283174 PMC3830948

[B81] Tsokas P, et al Conditional knockout of the PKC/PKMζ gene in the adult mouse hippocampus prevents L-LTP. in 2012 Society for Neuroscience Meeting: New Orleans 2012 SfN: LA

[B82] Petersen JD Distribution of Postsynaptic Density (PSD)-95 and Ca2+/Calmodulin-Dependent Protein Kinase II at the PSD J Neurosci 2003 23 35 11270 11278 14657186 10.1523/JNEUROSCI.23-35-11270.2003 PMC6741048

[B83] Hamilton AM Activity-dependent growth of new dendritic spines is regulated by the proteasome Neuron 2012 74 6 1023 30 10.1016/j.neuron.2012.04.031 22726833 PMC3500563

